# Development of a Feed Conversion Ratio Prediction Model for Yorkshire Boars Using Cumulative Feed Intake

**DOI:** 10.3390/ani15040507

**Published:** 2025-02-11

**Authors:** Hao Zhou, Haoshi Cheng, Yuyang Wang, Dongdong Duan, Jinyi Han, Shenping Zhou, Wenshui Xin, Xinjian Li

**Affiliations:** 1 College of Animal Sciences and Technology, Henan Agricultural University, Zhengzhou 450046, China; 13703822441@163.com (H.Z.); 18837401070@163.com (H.C.); yywang1009@163.com (Y.W.); 2 Sanya Institute, Hainan Academy of Agricultural, Sanya 572025, China; duandongd@126.com (D.D.); hjy7jane@126.com (J.H.); 18819427274@163.com (S.Z.)

**Keywords:** cumulative feed intake, feed conversion ratio, Yorkshire boars, Bayesian ridge regression

## Abstract

Feed conversion ratio (FCR) is a key indicator of pig productivity, but its measurement is labor-intensive and time-consuming. This study aimed to construct a predictive model for cumulative feeding intake (CFI), which could help estimate FCR more efficiently and reduce the time and effort needed for measurements. This study included a total of 987 Yorkshire boars raised in specific pathogen-free environments, with feeding and growth data collected using automatic feeders. The segmented R package and Bayesian ridge regression (BRR) were used to build a predictive model for CFI. The results showed that the optimal body weight range for predicting FCR was 80–110 kg. The BRR model achieved 80% accuracy for CFI prediction, and FCR calculated from predicted CFI showed 81.4% similarity to the corrected FCR. The results clearly demonstrate that even with a limited training dataset, the BRR model has good predictive potential for FCR. The findings of this study could reduce the selection pressure on FCR traits, decrease production costs, and shorten measurement periods, ultimately benefiting the swine industry significantly.

## Simple Summary

Feed conversion ratio (FCR) is crucial in pig breeding but labor-intensive to measure. This study explored the optimal body weight range (30–120 kg) using the segmented R package and developed a Bayesian ridge regression (BRR) model to predict cumulative feed intake (CFI) based on data from 987 Yorkshire boars. The 80–110 kg range was optimal for growth trait prediction, with the BRR model achieving 0.80 accuracy for CFI. The FCR derived from predicted CFI showed a correlation of 0.81 with the actual values. Additionally, we developed a CFI correction formula with an accuracy of 0.90. These findings provide a basis for improving feed efficiency assessment and reducing measurement costs.

## 1. Introduction

According to the statistics from the Food and Agriculture Organization (FAO), the grain utilized in livestock production constitutes a substantial portion of the agricultural gross value added. In particular, during the pig production process, feed costs account for more than 60% of the total production costs of pigs [1]; therefore, improving feed efficiency is one of the important ways to reduce the production cost of pigs. The feed conversion ratio (FCR) has been used in pig breeding for many years, but the progress remains slow. The fundamental reason is that the acquisition of FCR phenotypes is a time-consuming and economic process. Therefore, finding methods for efficiently obtaining the FCR phenotype has become an urgent problem to be solved in the current pig breeding industry [2,3].

With the increasing use of Automatic Feeding Systems (AFS), it has become feasible to assess individual pig FCR [4,5]. During pig production, each measuring device can collect thousands of data points, including feed intake (FI), feeding time (FT), and body weight (BW) [6,7]. The devices currently in use for pigs still exhibit certain general and individual errors, despite the high level of automation and intelligence in AFS, which significantly improves data collection efficiency. For instance, issues such as missing ear-tag readings, negative FI values, and unusually high single-feed intake measurements frequently occur [8]. These issues clearly contradict the normal growth patterns of pigs [9]. Therefore, many researchers are currently focused on accurately processing AFS data. Examples include the establishment of data quality control standards for AFS, incorporating linear mixed models for data processing, and employing multiple imputation (MI) and multiple imputation by chained equations (MICE) to estimate missing feed intake data [10]. Additionally, a segmented approach has been applied to extract phenotypic data from AFS [11], while methods such as quadratic regression, orthogonal polynomial regression, and locally weighted regression have been used to correct missing data [12], all of which can improve data accuracy and reliability to some extent. In the current research, multiple indices have been proposed to quantify FCR, such as the feed-to-gain ratio (F/G) [13] and residual feed intake (RFI) [14,15], which can to some extent reflect an individual pig’s FCR. However, variations in growth rates among pigs make it difficult to standardize these indices within a uniform body weight range, thereby limiting the comparability of individual FCR under the same evaluation framework. In numerous studies on FCR, data collection often demands considerable investment in terms of time and effort. Therefore, reducing the measurement period for pigs without compromising the accuracy of phenotypic calculations is critical for alleviating the burden of obtaining FCR data. Wang et al. [16] previously suggested reducing the measurement period for cattle from 112 days to 42 days. However, in current swine research, reducing the measurement period while accurately capturing pig FCR remains a significant challenge, and there is limited related research.

In this study, we meticulously processed feeding data from 987 pigs to obtain standardized FCR metrics. The objectives of this study are (1) to accurately process 30–120 kg FCR data, (2) to develop a predictive model for 30–120 kg FCR, and (3) to advance the genetic improvement of pig FCR. This study offers new insights for FCR studies and provides theoretical support for FCR trait selection in the swine industry.

## 2. Materials and Methods

### 2.1. Experimental Animals

This study involved a total of 987 boars, all reared under uniform feeding conditions with unrestricted access to water and feed. Throughout the study, all pigs remained healthy. Each measurement station housed 15 pigs, with individual feeding behavior and BW changes automatically recorded throughout the day using automatic feeders (https://osbornelivestockequipment.com, accessed on 2 January 2025). The feeders were maintained daily by trained personnel. All pigs were introduced to the measurement stations simultaneously, with an initial BW of 28.30 ± 1.02 kg, as determined at the time of entry. The ambient temperature in the pig housing was maintained at 18–22 °C to ensure optimal growth conditions. Feeding standards are specified in Table 1.

### 2.2. Data Collection and Quality Control

The initial phenotypic data for this study were collected from 987 pigs, resulting in a total of 764,254 raw data points, which included FT, BW, and FI. Of these, 755,486 data points contained complete information for all three traits (FT, FI, and BW). Following the quality control criteria established by Jiao and collaborators [10], the raw data were preprocessed based on three variables: feeding rate, single feeding amount, and feeding time, using eight quality control standards (Table 2). Anomalous data were excluded during preprocessing, leaving 746,443 valid records after the quality control.

### 2.3. Statistical Analysis

Robust regression is used to address the influence of outliers or deviations in data, thereby enhancing the reliability of the regression model. This method was employed to identify outliers in each pig’s BW data, with individuals having a Pseudo-R-squared value below 0.8 being excluded from the analysis. The slope derived from robust regression was used to represent each pig’s average daily gain (ADG). A three-parameter logistic growth model [17] was used to fit the data post-robust regression and predict the growth trends of the three breeds. The logistic growth model fitting formula is as follows:

$$BW=\frac{{BW}_{Max}}{{1+e}^{\frac{xmid-Day}{scal}}}$$

In the formula, BW represents the predicted body weight, BW_Max_ denotes the maximum capacity weight, which is the heaviest weight the animal is expected to reach. Xmid is the age at which half of the maximum weight is reached. Scal is the steepness of the curve.

For anomalies in the feeding data, individual single feeding amounts that deviated more than three standard deviations from the population mean were marked as outliers. A multiple regression model was constructed using feeding time and BW as variables to predict the FI. The correction formula is as follows:

FI=BW+T+BW:T

where FI is the predicted feed intake, BW is the measured weight during the feeding event, T is the feeding time (FT) for that event, and BW:T represents the interaction term between BW and feeding time, reflecting their combined effect on predicting FI.

### 2.4. Construction of the Correction Formula for the Cumulative Feed Intake of Pigs with Body Weight from 30 Kg to 120 Kg

The cumulative feed intake (CFI) was defined as the total feed intake accumulated by pigs over the measurement period. To obtain a reliable CFI curve, we selected CFI data along with weight data from 25 to 50 kg at the start of the measurement and from 100 to 140 kg at the end of the measurement. The lm function in R was used to fit a univariate regression equation between BW and CFI for each individual. We then excluded individuals with an R^2^ of less than 0.9, as these were likely to have anomalous feed intake data. The CF and A models were employed to estimate the correction coefficients for CFI_120_. The formulae for the CF and A models are as follows:

CFI120CF=CFIend−(BWend−120)×CFIend(BWend−BWin)×CF

CFI30CF=((30−BWin)×CFIend(BWend−BWin)×CF

CFI120A=CFIend+(120−BWend)×(CFIend−A)BWend

CFI30A=(30−BWin)×(CFIend−A)BWend

where CFI_120_ represents the corrected cumulative FI at 120 kg, CFI_end_ is the cumulative FI recorded at the end of the measurement period, and BW_in_ and BW_end_ are the BW at the start and end of the measurement. CF and A are the correction coefficients for the two models. CF is defined as the ratio of the slope of the best-fit line to the slope of the line connecting the starting and ending measurement points. The intercept of the linear regression model relating BW to CFI is denoted as A.

To assess correction accuracy, univariate regression equations for BW and CFI were used to calculate the regression-based CFI from 30 to 120 kg (CFI_reg_) for each individual, serving as an unbiased estimate of the true CFI_30–120_. The correlation coefficients between CFI_30–120CF,_ CFI_30–120A_, and CFI_reg_ were calculated using the cor.test function in R, with values closer to 1 indicating a better performance.

### 2.5. Construction of the Prediction Model

Data on pigs weighing 30 to 120 kg across the breeds were analyzed using sliding-window techniques with module sizes ranging from 10 to 80 kg and a module distance of 10 kg. Spearman correlation coefficients between the overall FCR and module FCR were calculated for each size, and the average correlation coefficient was used to represent each module. The breakpoints in the correlation coefficients were identified using the segmented function from the R package, with the module size at the breakpoint considered as the optimal measurement range. The Bayesian ridge regression (BRR) model was then used to predict the CFI for pigs in the 30–120 kg range, using the prediction model as follows:

$${CFI}_{p}=\beta 1\times {BW}_{in}+\beta 2\times {BW}_{out}+\beta 3*CFI+Intercept$$

where CFI_p_ represents the predicted FI for pigs within the 30–120 kg weight range, BW_in_ denotes the BW at the start of the measurement, BW_out_ represents the BW at the end of the measurement, CFI is the measured FI data during the measurement period, Intercept is the model intercept, and β1, β2, and β3 are the coefficients obtained from model training.

To validate the accuracy of the predicted FCR, the correlation coefficients between the predicted and true values were calculated using the cor.test function in R software package. The correlation coefficient serves as an indicator of predictive accuracy, with a higher coefficient reflecting greater accuracy. The predicted FCR for an individual pig during the prediction period can be obtained by dividing the predicted cumulative feed intake (CFI) by the measurement time within the predicted weight range.

### 2.6. Grouped Validation

To validate the CFI correction formula for the 30 to 120 kg range and evaluate the predictive performance of the BRR model for CFI, a grouped validation was conducted using data from 150 Yorkshire boars from different batches raised on the same farm. The correction formula was applied to adjust CFI values, followed by correlation analyses among the CFI_reg_, CFI_30–120CF_, and CFI_30–120A_. Daily feed intake (DFI) data for the 30–120 kg and 80–110 kg ranges were extracted to calculate the corresponding CFI values. Correlation coefficients were computed to assess the model’s accuracy by comparing FCR calculated from predicted CFI with actual FCR values, ensuring robust validation of both the correction formula and the predictive model.

## 3. Results

### 3.1. Descriptive Statistics

**Figure 1:**
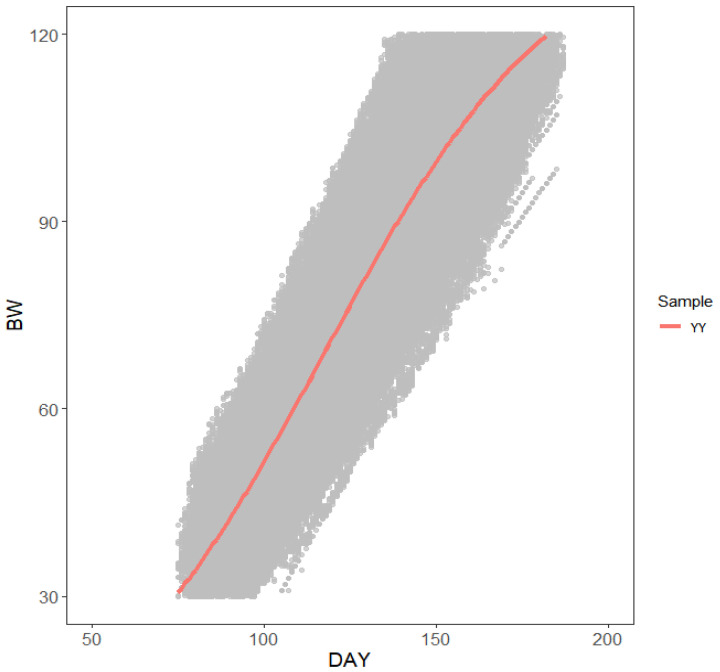
Fitting renderings of the logistic growth model for Yorkshire. In the figure, the orange line represents the Yorkshire breed. The *x*-axis denotes age in days of pigs after birth, and the *y*-axis denotes body weight of pigs.

In this study, we constructed a multiple regression model using FT and BW to correct for abnormal FI. The results of the FI correction indicated a marginal R-squared value of 0.87 and conditional R-squared value of 0.91. Furthermore, the calculated mean daily FI of Yorkshire boars was 2.35 kg/day. The robust regression analysis of individual animals revealed that the average R-squared value for the regression between BW and DAY was 0.962, with a maximum value of 0.997 and a minimum value of 0.801. After performing a robust regression analysis, BW data were used to construct a logistic growth model. The fitting results are shown in Figure 1. The modeled logistic curves showed that Yorkshire boar breeds exhibited the classic S-growth curve, with a growth rate characterized as “Slow-Fast-Slow”. The modeled logistic formula indicated that the number of days at which the Yorkshire populations reached their median BW was 117.17 days, with a corresponding BW of 63.67 kg. Phenotypic processing results are listed in Table 3.

### 3.2. Results of the Correction Formula Construction for 30–120 Kg

**Figure 2:**
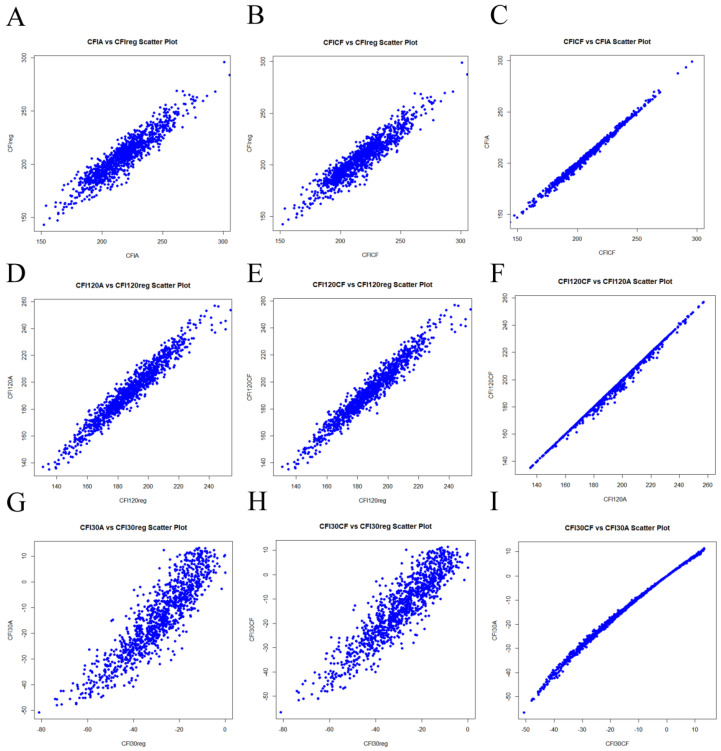
Scatter plots of CFI_A_, CFI_CF,_ and CFI_reg._ (**A**–**C**) illustrate the scatter plots for cumulative feed intake corrections between CFI_A_, CFI_CF_, and CFI_reg_ for the 30–120 kg range. (**D**–**F**) show the scatter plots for cumulative feed intake corrections at 120 kg versus regression-based cumulative feed intake. (**G**–**I**) present scatter plots for cumulative feed intake corrections at 30 kg versus regression-based cumulative feed intake. In the figures, blue points represent individual predictions and regression data.

**Figure 3:**
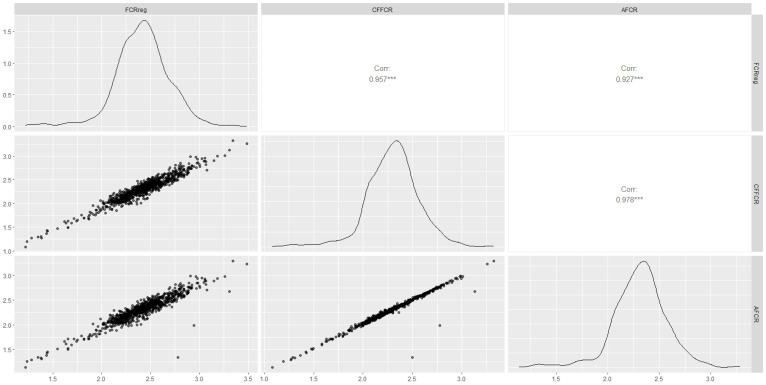
Pairwise matrix plot of the correlation between corrected FCR and true FCR in the validation population of 150 boars. The lower left corner shows scatter plots of AFCR and CFFCR versus the true FCR values. The diagonal presents density plots of AFCR and CFFCR versus true FCR values. The upper right corner displays the correlation coefficients and significance levels between AFCR, CFFCR, and true FCR values. *** indicates highly significant, *p* < 0.001.

The basic statistical summary of the correction coefficients is presented in Table 4. The CF correction coefficient was 1.03, and the correction coefficient A was −103.52. Scatter plots comparing the correction formula estimates with actual values are shown in Figure 2. Strong correlations were observed between the CFI_reg_ and both CFI_A_ (r = 0.93, *p* < 0.0001) and CFI_CF_ (r = 0.96, *p* < 0.0001) (Figure 2A,B), as well as between CFI_A_ and CFI_CF_ (r = 0.98, *p* < 0.0001). The average differences between CFI_reg_ and CFI_A_ and CFI_CF_ were 9.50 kg (SD = 9.05 kg) and 10.45 kg (SD = 8.06 kg). The scatter plots for CFI_120reg_ versus CFI_120A_ (r = 0.97) and CFI_120CF_ (r = 0.97) also showed strong correlations (Figure 2D,E). Similarly, CFI30_reg_ exhibited high correlations with CFI_30A_ (r = 0.90) and CFI_30CF_ (r = 0.91) (Figure 2G,H). Using the two correction formulas mentioned above to correct CFI, the calculated FCR results show that both corrected FCRs are highly correlated with the true FCR (Figure 3), with average errors of 0.11 (SD = 0.08) for AFCR and 0.11 (SD = 0.11) for CFFCR. The results indicate that the correction model demonstrates high accuracy, reaching approximately 92%.

**Figure 4:**
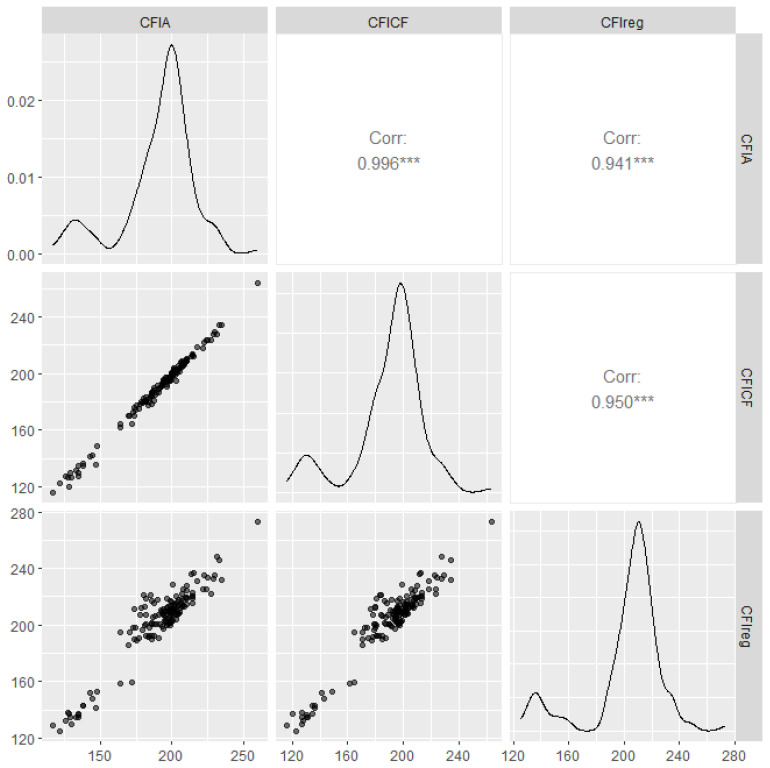
Pairwise matrix plot of the correlation between corrected CFI and CFI_reg_ in the validation population. The lower left corner shows scatter plots of ACFI and CFCFI versus the CFI_reg_. The diagonal presents density plots of ACFI and CFCFI versus CFI_reg_. The upper right corner displays the correlation coefficients and significance levels between ACFI, CFCFI, and CFI_reg_ in the validation population. *** indicates highly significant, *p* < 0.001.

The validation results for the correction formula in the validation group are shown in Figure 4. In the new group, the correlation coefficients between the corrected CFI_CF_, CFI_A_, and CFI_reg_ were 0.95 and 0.94. This further validates the feasibility of the model.

### 3.3. Predictive Model for Cumulative Feed Intake in the 80–110 Kg Range

**Figure 5:**
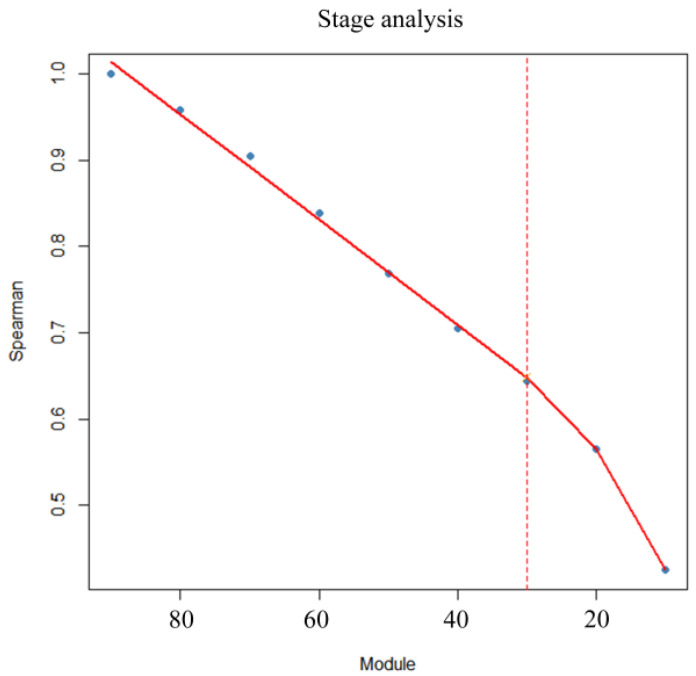
Stage division results. The *x*-axis represents the weight range within each module, while the *y*-axis represents the average Spearman correlation coefficient between the FCR under each module and the FCR for the weight range of 30–120 kg.

**Figure 6:**
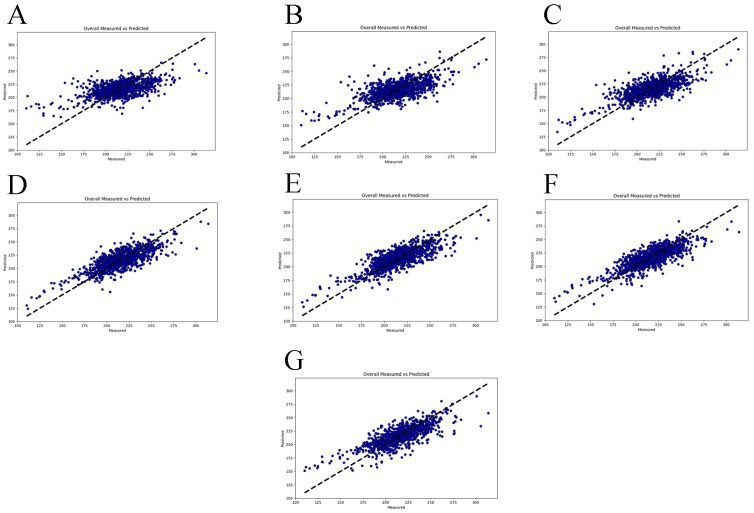
Scatter plots of module-predicted CFI vs. CFI_reg._ (**A**–**G**) display scatter plots of predicted CFI values versus CFI_reg_ values for different modules. Each subplot represents a specific module size: 30–60 kg, 40–70 kg, 50–80 kg, 60–90 kg, 70–100 kg, 80–110 kg, and 90–120 kg, respectively. The horizontal axis represents the CFI_reg_ values, and the vertical axis shows the CFI values predicted by the module.

The stage division results for the FCR across different measurement periods are depicted in Figure 5, highlighting a critical inflection point in the feed conversion efficiency at 30 kg. Consequently, a module size of 30 kg was selected to predict the FCR from 30 kg to 120 kg. The prediction outcomes, shown in Figure 6, indicate that the model performed most effectively within the 80–110 kg range. To further validate the model accuracy, five-fold and ten-fold cross-validation was conducted for each module (Table 5). The results demonstrated that the BRR model achieved optimal performance using CFI data from the 80–110 kg range to predict CFI for the 30–120 kg range, with an average MSE of 204.44 ± 30.67, RMSE of 14.26 ± 1.09, and R^2^ of 0.65 ± 0.06. Correlation coefficients of Spearman (0.80) and Pearson (0.83) correlation coefficients between the predicted CFI and CFI_reg_ in the 80–110 kg module were the highest among all the modules. The correlation coefficient between the predicted and actual FCR was calculated to assess the reliability of the predicted CFI, yielding a Spearman correlation of 0.81. The results indicate that the predictive model demonstrates high accuracy, achieving over 80% in predicting CFI.

**Figure 7:**
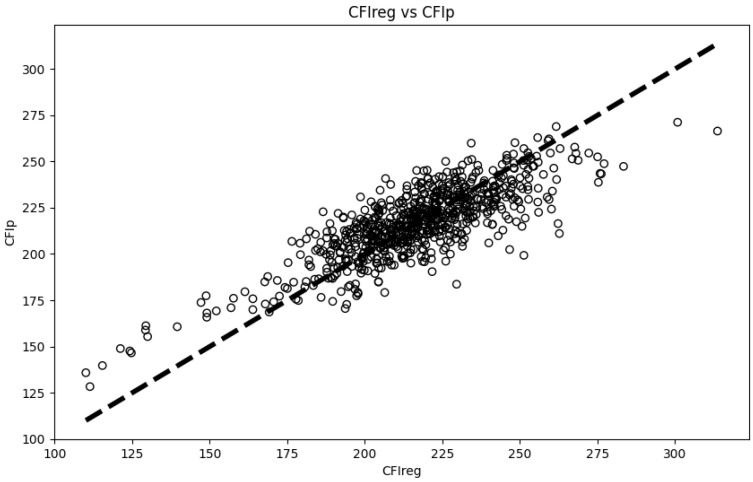
Scatter plots of CFI_P_ and CFI_reg_ in the validation population. The *x*-axis represents the true unbiased estimates of CFI, while the *y*-axis represents the predicted values of CFI.

The BRR model was applied to the validation group, and the results are shown in Figure 7. The five-fold and ten-fold cross-validation produced an average MSE of 204.44 ± 30.67, RMSE of 14.26 ± 1.09, and R^2^ of 0.65 ± 0.06. The coefficients between the CFI_P_ and CFI_reg_ were 0.70 for Spearman and 0.79 for Pearson correlation. For the FCR, the predicted and actual values had coefficients of 0.70 for Spearman and 0.79 for Pearson correlation. This further validates the feasibility of the model.

## 4. Discussion

Data related to pig feeding behavior and FCR often rely on AFS systems. However, collecting data using AFS often results in challenges, such as a high volume of abnormal data, lengthy measurement periods, and significant labor demands. Therefore, maximizing the efficient use of AFS-generated data is crucial for improving FCR selection in the swine industry [18].

Prior to this study, numerous investigations focused on the phenotypic processing of FCR. However, most of these studies applied only basic quality control and linear imputation for abnormal BW and FI data [19,20]. Given the characteristics of FCR data, which typically involve extended collection periods and large data volumes, traditional quality control methods and simple linear imputation are insufficient for accurately reflecting FCR in pigs [21]. This limitation has been a significant factor contributing to the slow progress in the genetic improvement of FCR-related traits in pigs. Numerous scholars have proposed valuable methodologies for processing FCR data. For instance, Williard C. Losinger utilized the MIXED procedure in SAS to perform maximum likelihood estimation of FCR and related traits [22]. In this study, a robust regression model was employed to correct abnormal BW data. This statistical method is specifically designed to enhance the resilience of regression models by addressing outliers and deviations, proving more effective than ordinary linear models in mitigating the influence of such anomalies [23]. Additionally, a logistic model was applied to validate the corrected BW data. The results demonstrated that the growth trend of Landrace pigs follows an “S-shaped” slow-fast-slow curve, consistent with the findings of Veylit L. and colleagues [24], thereby affirming the reliability of our approach. Of course, some researchers have also attempted to correct FI, a trait highly correlated with FCR, using various models. For example, H. Nguyen-Ba [25] and colleagues proposed a method to quantify FI in growing pigs. Although many researchers have proposed various methods for processing FI data, these approaches have not achieved the standardization necessary to directly compare FI and FCR. Therefore, establishing a unified standard for FCR comparison remains an urgent challenge. To the best of our knowledge, this aspect has not been addressed in prior studies, leaving a significant gap in the research. Through our research, we identified a univariate linear relationship between CFI and BW, similar to the correction formulas for pig age at 100 kg established by the Canadian Swine Improvement Program and the National Swine Improvement Federation in the U.S. Based on these formulas [26,27], we successfully developed the A model and CF model in this study. After validation using both the test and validation populations, the models achieved an accuracy of over 95%. These findings are consistent with those of Zheng Hao and collaborators [26], who constructed a similar correction formula for the age at 100 kg in Licha Black pigs, further demonstrating the feasibility of the correction formulas proposed in this study.

In the current swine industry, determining feed-related traits often requires substantial investment in labor and resources [28]. In previous studies, obtaining FCR data typically required a collection period of over 90 days, generating thousands of data points [4,29]. This extensive time frame and data volume have been major challenges in the selection of FCR traits. For example, Chris Davison and collaborators predicted FCR in cattle by applying a Support Vector Regression model to feed intake data collected from 80 beef cattle over a 56-day period [30]. FCR is a ratio trait that relies on the phenotypic measurement of two distinct components: ADG and ADFI. This intrinsic complexity makes direct prediction of FCR particularly challenging. Additionally, data collected through AFS frequently include errors, with the majority of inaccuracies concentrated in FI measurements. Such errors complicate the direct modeling of FCR and highlight the need for more robust prediction strategies. Given this context, leveraging stage-specific FI data to estimate CFI over the growth period offers a more practical and accurate approach [25]. By predicting CFI, it becomes feasible to indirectly estimate FCR with greater reliability. This method not only addresses the inherent challenges of FCR prediction but also mitigates the impact of erroneous FI data, ultimately providing a more consistent and interpretable framework for evaluating FCR in pigs.

This study revealed a univariate linear relationship between CFI and BW. Consequently, from the perspective of model construction complexity, using BW as the basis for stage division is a simpler and more practical approach. Compared to traditional linear regression and time-series analysis, BRR offers the advantages of Bayesian inference and ridge regression regularization [31]. This method automatically incorporates regularization during the estimation process, yielding posterior distributions for the parameters and avoiding overfitting, thereby providing more accurate estimates [32,33]. In previous studies, the BRR model has been widely applied to predict various metrics across different fields due to its effectiveness in handling complex, high-dimensional data [34,35]. For example, A. Ferragina and colleagues, in their work on developing a model to predict milk composition, found that the BRR model demonstrated superior predictive performance compared to Partial Least Squares regression [36]. This advantage was attributed to BRR’s ability to incorporate regularization, which helps in managing multicollinearity and preventing overfitting, thus improving the model’s accuracy [37,38]. Similarly, Mohd Saqib successfully applied the BRR model to predict the progression of the COVID-19 outbreak [39]. By incorporating key epidemiological factors into the model, Saqib was able to forecast the development of the pandemic with a high degree of accuracy. This demonstrated the BRR model’s versatility and effectiveness in predicting complex, time-dependent phenomena, further highlighting its potential in diverse applications ranging from agricultural research to public health [40,41]. The segmented package in R fits piecewise regression models, identifying breakpoints where the rate of change in the dependent variable shifts. It provides a robust framework for detecting and modeling potential breakpoints in linear, nonlinear, and generalized linear models [42]. In this study, the breakpoint module size was selected as the optimal measurement range because, after the breakpoint, the FCR correlation coefficient increased at a constant rate with the measurement period. The correlation coefficient exceeded 0.6 at the breakpoint, accurately reflecting individual pig FCR. Therefore, the breakpoint was chosen as the optimal measurement period. In studies concerning FCR, the majority of researchers typically select 20–30 kg as the starting BW and 100–120 kg as the ending BW for data collection [4,29]. This standard approach leads to a prolonged data collection period, often exceeding 100 days, to capture sufficient information on FCR. However, the results of this study, based on both stage division and the fitting outcomes of the BRR model, suggest a more efficient strategy. Specifically, utilizing CFI data from the 80–110 kg weight range to predict CFI data for the broader 30–120 kg range resulted in significantly more accurate predictions. This approach not only shortens the required data collection period but also improves the precision of FCR prediction. In both the test and validation populations, the predictive model was able to achieve a prediction accuracy of 70%, indicating that the 80–110 kg weight range offers a more reliable basis for estimating FCR. By focusing on this narrower weight range, the study demonstrates that it is possible to effectively predict FCR with high precision, reducing the need for extensive data collection over longer periods, which is typically a major bottleneck in FCR-related research. This result allows the measurement period to be reduced from the conventional minimum of 100 days to just 30–40 days, significantly improving the efficiency of the AFS. By shortening the data collection timeline, the study not only alleviates the pressure of obtaining FCR and related traits but also enhances the feasibility of incorporating these measurements into swine breeding programs. This advancement is particularly beneficial for the breeding industry, as it facilitates more efficient and timely selection for FCR and associated traits, ultimately supporting genetic improvement efforts in pigs with greater precision and less resource investment.

## 5. Conclusions

In this study, we developed a correction method for abnormal FI and constructed a BRR model to predict CFI in pigs. The correction formula demonstrated high precision, with R^2^ values exceeding 0.9 and strong correlations between corrected and actual FI values. The BRR model showed optimal performance within the 80–110 kg range, achieving an average R^2^ of 0.65 and high correlation coefficients between predicted and actual CFI and FCR values. Validation confirmed the model’s robustness and generalizability. These findings provide a precise and practical framework for phenotyping feed efficiency traits in pigs.

## Figures and Tables

**Table 1:** Standards for feeding.

Growth Stage	25–50 kg	50–75 kg	75-Marketed
Corn 8.2 ^(1)^	653.4	652.6	668.1
Wheat bran	70	70	132.9
Soybean meal	139.9	223.4	160
Extruded full fat soybeans	80	/	/
Soybean oil	12.7	10	/
Kim Tsai Fook ^(2)^	40	/	/
King Jin Yu ^(2)^	/	40	35
Premix Small Feed A ^(3)^	4	4	4

^(1)^ Corn 8.2 refers to the protein content of the corn being 8.2%. ^(2)^ Kim Tsai Fook and King Jin Yu are both complete feeds. ^(3)^ The premix method for Small Feed A involves adding 200 g of phytase per ton, mixing 50 kg of phytase with 950 kg of corn powder in a mixer for 90 s, and then removing and setting it aside for later use.

**Table 2:** Quality control standards for raw data.

Error Index	Error Type	Error Definition
1	FIV ^(1)^-low	FIV < −0 g for all visits
2	FIV-high	FIV > 2000 g for all visits
3	FIV-0	FIV > 20 g or FIV < −20 g for visits with OTV = 0 s
4	OTV ^(2)^-low	OTV < 0 s for all visits
5	OTV-high	OTV > 3600 s for all visits
6	FRV ^(3)^-high-FIV-low	FRV > 500 g/min for visits with 0 < FIV < 50 g
7	FRV-high	FRV > 350 g/min for visits with FIV > 50 g
8	FRV-0	FRV = 0 g/min for visits with OTV > 500 s

^(1)^ FIV = feed intake per visit (g); ^(2)^ OTV = occupation time per visit (s); ^(3)^ FRV = feed intake rate per visit (g/min).

**Table 3:** Summary statistics of the analyzed traits.

Traits	Abbreviations	Units	Yorkshire		
			N ^(1)^	Mean	SD ^(2)^
Average daily gain from 30 to 120 kg	ADG	kg	987	1.02	0.12
Average daily feed intake	ADFI	kg	987	2.35	0.32
Average occupation time in feeder per day	AOC	min	987	53.99	10.49
Average number of visits to feeder per day	AVT	counts	987	7.93	3.07
Average feed intake per visit	FPV	kg	987	0.25	0.25
Average occupation time in feeder per visit	OPV	min	987	5.67	5.26
Feed intake rate	FR	g/min	987	45.65	23.94
Feed conversion ratio	FCR	kg/kg	987	2.31	0.27

^(1)^ N = number. ^(2)^ SD = standard deviation.

**Table 4:** Simple statistics of the correction coefficients.

Type	N ^(1)^	Mean	SD ^(2)^	Min	Max
CF	815	1.03	0.04	0.87	1.20
A		−103.52	26.46	−301.02	−49.70

^(1)^ N = number. ^(2)^ SD = standard deviation.

**Table 5:** Five-fold ten-fold cross-validation results of the CFI prediction model.

Module	Average MSE	Average RMSE	Average R^2^	Spearman	Pearson
30–60	338.71 ± 39.01	18.37 ± 1.07	0.43 ± 0.04	0.49	0.56
40–70	271.19 ± 17.18	16.46 ± 0.53	0.54 ± 0.05	0.57	0.66
50–80	256.47 ± 16.68	16.01 ± 0.51	0.56 ± 0.06	0.64	0.71
60–90	230.59 ± 39.59	15.13 ± 1.27	0.61 ± 0.07	0.72	0.78
70–100	223.78 ± 21.67	14.94 ± 0.70	0.62 ± 0.07	0.74	0.80
80–110	204.44 ± 30.67	14.26 ± 1.09	0.65 ± 0.06	0.80	0.83
90–120	223.68 ± 33.33	14.91 ± 1.11	0.62 ± 0.03	0.76	0.78

## Data Availability

The original contributions presented in this study are included in the article. Further inquiries can be directed to the corresponding authors.
